# Natural killer cells: a promising immunotherapy for cancer

**DOI:** 10.1186/s12967-022-03437-0

**Published:** 2022-05-23

**Authors:** Junfeng Chu, Fengcai Gao, Meimei Yan, Shuang Zhao, Zheng Yan, Bian Shi, Yanyan Liu

**Affiliations:** 1 Department of Medical Oncology, The Affiliated Cancer Hospital of Zhengzhou University, Zhengzhou, 450008 Henan China; 2 Department of Hematology, The First Affiliated Hospital of Zhengzhou University, Zhengzhou, 450052 Henan China; 3 Department of Chinese and Western Medicine, The Affiliated Cancer Hospital of Zhengzhou University, Zhengzhou, 450008 Henan China

**Keywords:** NK cells, Immunotherapy, CAR-NK cells, Cancer

## Abstract

As a promising alternative platform for cellular immunotherapy, natural killer cells (NK) have recently gained attention as an important type of innate immune regulatory cell. NK cells can rapidly kill multiple adjacent cancer cells through non-MHC-restrictive effects. Although tumors may develop multiple resistance mechanisms to endogenous NK cell attack, in vitro activation, expansion, and genetic modification of NK cells can greatly enhance their anti-tumor activity and give them the ability to overcome drug resistance. Some of these approaches have been translated into clinical applications, and clinical trials of NK cell infusion in patients with hematological malignancies and solid tumors have thus far yielded many encouraging clinical results. CAR-T cells have exhibited great success in treating hematological malignancies, but their drawbacks include high manufacturing costs and potentially fatal toxicity, such as cytokine release syndrome. To overcome these issues, CAR-NK cells were generated through genetic engineering and demonstrated significant clinical responses and lower adverse effects compared with CAR-T cell therapy. In this review, we summarize recent advances in NK cell immunotherapy, focusing on NK cell biology and function, the types of NK cell therapy, and clinical trials and future perspectives on NK cell therapy.

## Introduction

Globally, cancer is a great threat to human health and one of the leading causes of death [1]. For decades, surgery, chemotherapy, and radiotherapy have been the main methods of treating tumors in patients [2]. Nevertheless, the development of resistance to chemotherapy and/or radiotherapy is associated with a high incidence of cancer recurrence [3–5]. Treatment of these cancers can reduce physical strength and impair immune response, which can lead to recurrence as well as metastasis of remaining tumor cells in the body after treatment. Therefore, scholars should urgently uncover novel strategies for eliminating these resistant cancer cells. For many years, immune cells have been demonstrated to be significant targets for cancers. By 1984, immunotherapy was considered the fourth therapy after surgery, chemotherapy, and radiotherapy [6].

Immune cells roughly confer innate and adaptive immunity, which actively avert cancer development through immunosurveillance [7–10]. Innate immune cells consist of natural killer (NK) cells, dendritic cells (DCs), monocytes, and macrophages [11, 12]. These cells mediate the release of cytokines through an immediate and short-lived immune response. Cytokines then drive the following processes: (i) the direct lysing of cancer cells or the capture of dead cancer cells; (ii) antigen processing performed by phagocytose cancer cells; (iii) the activation of T cells mediated adaptive anti-tumor immune responses; and (iv) the release of cytoplasmic granules containing perforin and granzymes, which directly kill cancer cells and so on [13–17]. T cells and B cells constitute the adaptive immune cells, which are responsible for long-lived, antigen-distinct reactions and effective immune memory [13].

**Fig. 1:**
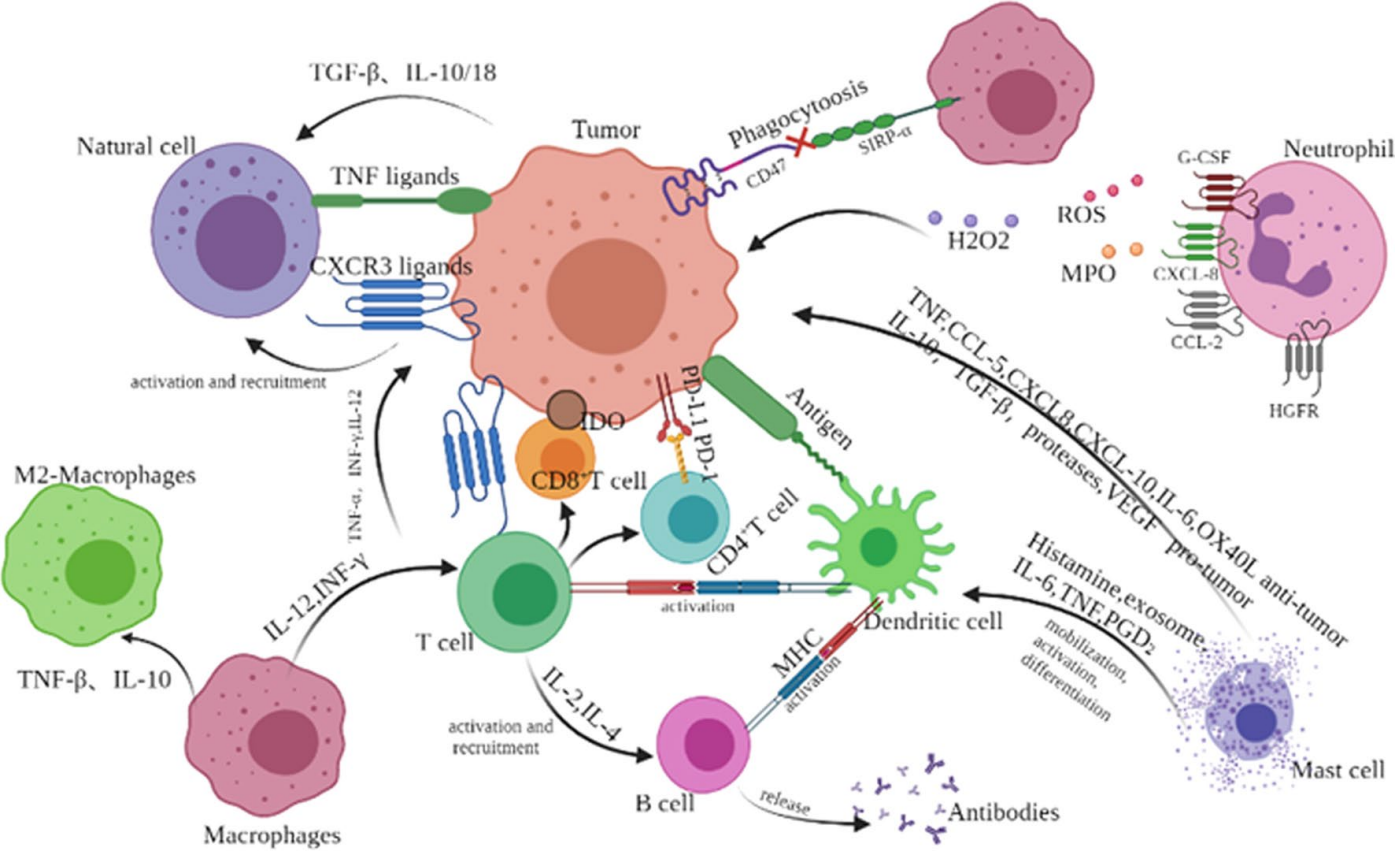
Schematic diagram of tumor-infiltrating immune cells interactions among each other and with cancer cells. Innate immune response cells (macrophages, mast cells and neutrophils) and adaptive immune response cells (lymphocytes) interact with tumor cells through chemokines, adipose cytokines and cytokines

Despite innate and adaptive immune reactions in our body, cancer cells can evade immunosurveillance through several mechanisms [18–24] (Fig. 1). For instance, they secrete the immunosuppressive cytokines TGF-β and IL-10, which repress the adaptive anti-tumor immune response [25, 26] or polarize tumor-associated macrophages (TAMs) toward an M2 phenotype that has significantly less anti-tumor potential but highly promotes tumor growth and metastasis capacity [27]. Some cancers affect the release of IL-6 [28], IL-10 [29], VEGF [30], or GM-CSF [31] and impair the functions of DC either by inactivating or suppressing maturation. Some tumors induce T regulatory cells to repress tumor-distinct T cell reactions [18]. Some cancers also induce the expression of PD-L1, thereby exhausting T cells through interaction with PD-1 [32]. In normal conditions, a “don’t eat me” signal is expressed by erythroblasts that avoid phagocytosis by macrophages, whereas senescent red blood cells cease to have the ability to express CD47 and are engulfed by macrophages [33–35]. Other cancers also express CD47 to avoid phagocytosis by macrophages in the tumor microenvironment (TME) [27, 36, 37]. Furthermore, malignant cells express minimal levels of tumor-associated antigens, dislodge NK cell-activating receptor ligands, or alter the expression of MHC-I as well as costimulatory biomolecules to escape immune reactions [13, 38].

Immunotherapies are developed to trigger either a prospective active or passive anti-tumor reaction against cancers by activating immune responses [39, 40]. To date, several cancer immunotherapies have been used in clinical practice, such as cytokines [10, 41, 42], monoclonal antibodies [43], vaccines [44–46], adoptive cell transfer (T [47–52], DC [53–55], NK [56, 57], and NK-T [58, 59]), and toll-like receptor (TLR) agonists [60–62]. In particular, NK cell immunotherapy has remained highly promising for over 30 years. Recently, in the NK cell biology field, remarkable advancements have been made in the comprehension of the function of NK cells as an effective cancer immunotherapy tool. Notably, NK cell therapy has been subjected to clinical phase I/II trials. In this paper, we review recent advancements in NK cell immune therapy, with an emphasis on the biology of NK cells, the function and types of NK cell therapy, and clinical trials as well as future perspectives on NK cell therapy.

## Development, classification, and distribution of NK cells

**Fig. 2:**
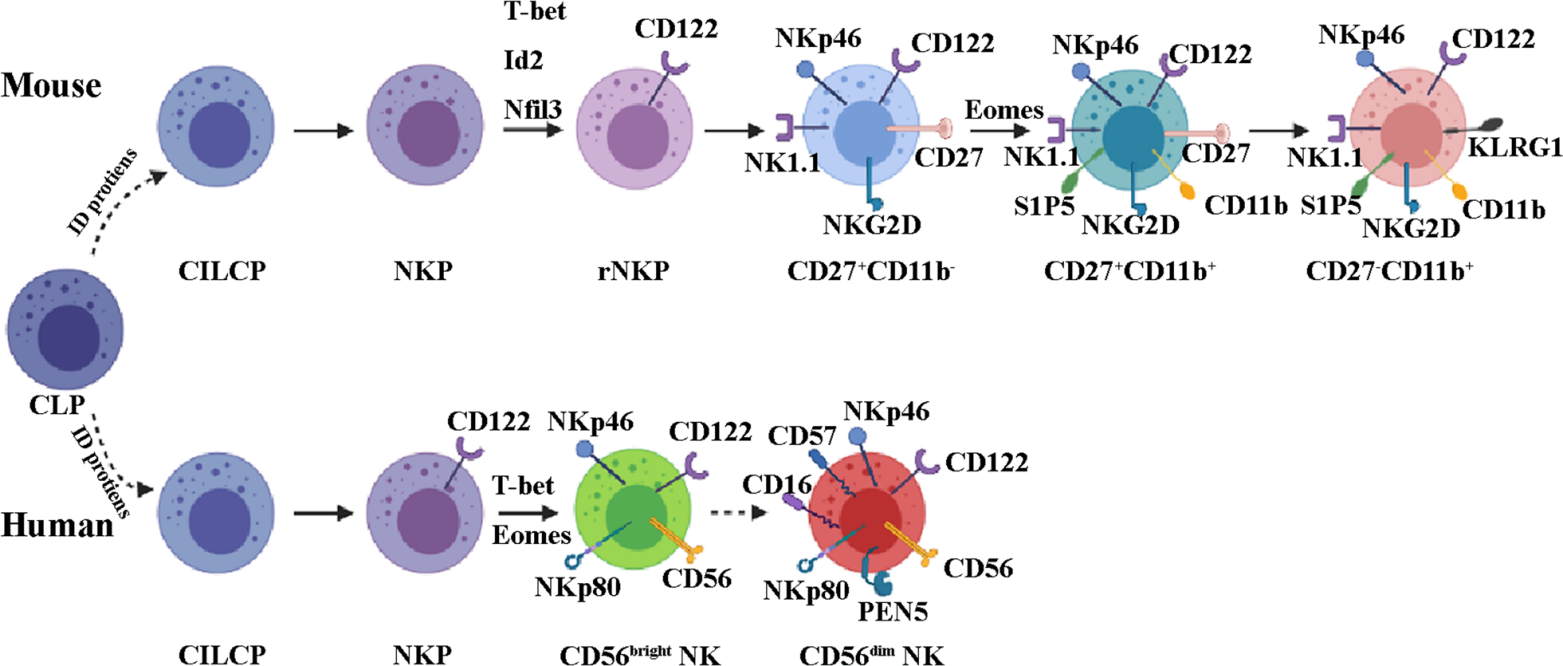
NK cell development. In mice, common lymphoid progenitor (CLP) produces common ILC precursor, CILCP). CILCP can produce NK cells and helper-like ILCs. There are at least five other stages in NK cell development from CILCP: NK progenitor cells (NKP), refined-NKP (rNKP), CD27^+^CD11b^−^NK, CD27^+^CD11b^+^NK and CD27^−^CD11b^+^ NK. In humans, after CILCP is developed from CLP, NK-restricted NKP will be developed from the latter. NKs is characterized by expressing CD122, losing CD34 and CD127. The expression of T-bet and Eomes is needed for further differentiation into functional NK cells. The expression of CD56 can divide NK cells into two subgroups: CD56^dim^ and CD56^bright^. The two subsets express activated surface receptors NKp46 and NKp80. CD56^+^ NK cells can differentiate into CD56^−^ NK cells by expressing CD16, PEN5 and CD57

NK cells are unique lymphocyte subpopulations that are larger than T as well as B lymphocytes and contain unique cytoplasmic granules [63]. They were first identified and discovered in 1975 by Herberman et al. [64] and Kiessling et al. [65]. NK cells originate from CD34^+^ hematopoietic progenitor cells in a continuous process in which common lymphocyte progenitors (CLPs) steadily downregulate CD34 while upregulating CD56. These events prompt the differentiation of NK cells along with their maturation [66–69]. In humans, a pluripotent progenitor cell close to CLPs has the potential to produce all subpopulations of ILCs. ILC-restricted common ILC precursor (CILCP) arises from CLP and subsequently generates the NK-restricted NKP. NKs are associated with CD122 expression and the loss of CD34 and CD127. Notably, the expression of T-bet and Eomes is required for the further differentiation of functional NK cells [70–72]. In mice, CLP cells produce CILCP, which successively generates NK cells as well as helper-like ILCs. Beginning with CILCPs, the development of NK cells comprises at least five phases: NKP → rNKP → CD27^+^CD11b^−^NK → CD27^+^CD11b^+^NK → CD27^−^CD11b^+^NK [73, 74] (Fig. 2).

Since NK cells express many surface markers, unique approaches exist for classifying distinct subsets of NK cells [70, 75, 76]. Based on CD56 expression, they are categorized into two cell subsets, namely CD56 ^low/dim^ and CD56^bright^. In particular, CD56^low/dim^ NK cells are anti-tumor cytotoxic, but both subsets can secrete cytokines [57, 77, 78]. NKp46- and NKp80-activating surface receptors are expressed in both NK cell subpopulations [79–81]. CD56^bright^ NK cells specialize into CD56^dim^ NK cells by expressing CD16, and PEN5 [82, 83]. Similarly, distinct subsets categorized based on their expression of CD11b, CD226 (DNAM-1), CD27, and KLRG1 have been studied in mice [84, 85]. However, no equivalence has thus far been established between the subsets of human NK cells and those of mice [86].

NK cells are extensively distributed throughout lymphoid as well as nonlymphoid tissues, consisting of the BM, liver, lungs, lymph nodes (LNs), spleen, liver, and peripheral blood (PB) [87, 88]. Studies have also found NK cells in several other organs, including the uterus, intestines, skin, adipose tissue, bladder, thymus, tonsils, kidneys, pancreas, and brain of mice [88]. In general, the CD56^low/dim^ subpopulation is dominant in human blood, whereas the CD56^bright^ subset is more dominant in lymph nodes [13, 89].

## The activation and inhibition of NK cells

### Inhibitory and activating signals

Several repressive and activating signal molecules are secreted by NK cells. At steady state, the suppressive receptors prevent the activation of NK cells and their subsequent killing effects. NK cells identify target cells in a non-MHC-restrictive manner, which then recognize the MHC-I molecule expressed on the intended cells. This recognition represses the stimulation of NK cells and prevents them attacking the body [13, 89–92]. Under stress, target cells diminish MHC-I expression. Consequently, NK cells lose repressive signaling and are activated through the “missing-self recognition” process [13, 89–95]. Some non-MHC biomolecules, such as Clr-b, CD48, and LLT-1 identified by the repressive receptors NKR-P1B [96], 2B4 [97], and NKR-P1A [98], respectively, also carry out the activation role. Other inhibitory receptors are killer cell immunoglobulin-like receptors (KIRs; e.g., KIR2DL and KIR3DL) [99] and c-type lectin receptors (CD94/NKG2A/B) [100]. Table 1 presents the inhibitory receptors associated with NK cells in a steady state. Accumulating evidence suggests that NK cells express activating receptors with potential recognition of either pathogen-encoded molecules or self-expressed proteins [13]. Under normal conditions, pathogen-coded biomolecules are not secreted by the host, and such recognition is termed “non-self-recognition” [13]. Additionally, disease-infected or transformed cells have been found to upregulate self-produced proteins. This phenomenon has been termed "stress-triggered self-recognition." [13]. Stimulating receptors comprise the cytotoxicity receptors NKp44 [101], NKp46 [79], and NKp30 [102]; the C-type lectin receptors NKG2E/H [103], CD94/NKG2C [104, 105], NKG2F [103], and NKG2D [106]; and the KIRs KIR-3DS and KIR-2DS [99], whereas repressive receptors comprise CD94/NKG2A/B and the KIRs KIR-2DL and KIR-3DL [99, 100]. Table 2 presents the activating receptors associated with NK cells in a steady state. Collectively, NK cells recognize their targets through the identification of numbers of stimulating as well as suppressive signals, whose outcomes depend on the nature of the target cells.

**Table 1:** The inhibitory receptors of NK cells

Receptors	CD	Structure	Ligand	Signal molecule	Chromosome
KIR2DL1	CD158b	Ig monomer	HLA-C, N77/N80	ITIM	19q13.4 [97]
KIR2DL2	CD158b1	Ig monomer	HLA-C, S77/N80	ITIM	19q13.4 [97]
KIR2DL3	CD158b2	Ig monomer	HLA-C, S77/N80	ITIM	19q13.4 [97]
KIR3DL1	CD158e1	Ig monomer	HLA-Bw4	ITIM	19q13.4 [105]
KIR3DL2	CD158k	Ig monomer	HLA-A3, HLA-A11	ITIM	19q13.4 [106]
KIR2DL5A/B	CD158f	Ig monomer	Unknown	ITIM	19q13.4 [107]
LAIR-1	CD305	Ig monomer	Collagen	ITIM	19q13.4 [107]
LILRB1(ILT2)	CD85j	Ig monomer	HLA-I	ITIM	19q13.4 [108]
SIGLEC7(p75)	CDw328	Ig monomer	Α-2,8 disialic acid	ITIM	19q13.3 [109]
CEACAM1	CD66a	Ig monomer	CD66	ITIM	19q13.2 [107]
CD94-NKG2A(KLRD1-KLRC1)	CD159a	C lectin heterodimer	HLA-E	ITIM	12p13 [110]
KLRG1		C lectin heterodimer	Cadherins	ITIM	12p12-p13 [107]
NKR-P1A(KLRB1)	CD161	C lectin heterodimer	LLT-1	ITIM	12p13 [107]
2B4	CD224	Ig monomer	CD48	ITIM	1q23.1 [107]

**Table 2:** The activating receptors of NK cells

Receptors	CD	Structure	Ligand	Signal molecule	Chromosome
2B4	CD224	Ig monomer	CD48	ITSM, SAP	1q23.1 [107]
KIR2DS1	CD158h	Ig monomer	HLA-C, N77/N80	DAP12	19q13.4 [97]
KIR2DS2	CD158j	Ig monomer	Unknown	DAP12	19q13.4 [111]
KIR2DS4	CD158i	Ig monomer	HLA-Cw4	DAP12	19q13.4 [66]
KIR3DS1	CD158e2	Ig monomer	Unknown	DAP12	19q13.4 [112]
KIR2DL4	CD158d	Ig monomer	HLA-G	FcεRIγ	19q13.4 [113]
NKp46(NCR1)	CD335	Ig monomer	HV	FcεRIγ, CD3ζ	19q13.4 [78]
NKp44(NCR2)	CD336	Ig monomer	HV	DAP12	6p21.1 [100]
NKp30(NCR3)	CD337	Ig monomer	Pp65, BAT-3, B7-H6	FcεRIγ, CD3ζ	6p21.3 [100]
FCGR3(FcγRIII)	CD16	Ig monomer	IgG	FcεRIγ, CD3ζ	1q23 [114]
DNAM-1	CD226	Ig monomer	CD112, CD155	Protein kinase C	18q22.3 [115]
SLAMF7	CD319	Ig monomer	CRACC	ITSM, EAT2	1q23.1–4 [116]
SLAMF6	No	Ig monomer	NBT-A	ITSM	1q23.2 [117]
TACTILE	CD96	Ig monomer	CD112, CD155	Unknown	3q13-q12.2 [118]
CD27	CD27	Ig monomer	CD70	TRAF2, TRAF5, SIVA	12p13 [119]
CD94-NKG2C(KLRD1-KLRC2)	CD159c	C lectin heterodimer	HLA-E	DAP12	12p13 [120]
CD94-NKG2E	No	C lectin heterodimer	HLA-E	DAP12	12p13 [120]
NKG2D(KLRK1)	CD314	C lectin heterodimer	ULBP1-4, MICA/B	DAP10	12p13 [87]
NKp80(KLRF1)	No	C lectin heterodimer	AICL	Unknown	12p13.2-p12.3 [79]

### Regulatory cytokines that increase NK cells in the TME

Ongoing research on NK cells relays increasing evidence regarding the critical roles they play in the early regulation of viral infection, in HSC transplantation (HSCT; improved grafting, graft vs. tumor effect, and graft vs. host disease), and in cancer immune surveillance among others [107]. Several regulatory cytokines induce the functions of NK cells as tumor targets [108]. Numerous cytokines (IL-2, IL-21, IL-12, IL-18, and IL-15) and type I interferons can be adopted for in vitro multiplication along with the induction of NK cells prior to adoptive transfer. Notably, individual activating receptors trigger cytokine secretion or insufficient cytotoxicity in naïve NK cells. Exposure to cytokines plays a remarkable role in the preactivation of NK cells. IL-12 enhances signaling from the activating receptors of NK cells [109]. IL-12 combined with IL-15 and IL-18 is especially attractive since it induced a memory-like NK cell population, which grew in immune-incompetent mice inoculated with exogenous IL-2 [110]. Patients transfused with NK cells are frequently given IL-2 to promote in vivo expansion [111]. One study found that repeated administration of IL-2 at low doses was well tolerated; however, no clinical benefit of IL-2 treatment was reported in a corresponding-pairs assessment [112]. The anti-malignant influences of IL-12 and IL-18 as mono-agents are quite minimal [108]. By contrast, IL-21 is potent, particularly in combination with cancer-targeting monoclonal antibodies (mAbs) [113]. The IL-15 cytokine is the one that demonstrates the best promise, with advancements being made in establishing IL-15 signaling mechanisms in NK cells [108]. A phase I clinical trial involving individuals with metastatic cancers found that daily administration of IL-15 induced the proliferation of NK cells and elevated NK cell numbers substantially [114]. Even though no objective response was reported in the trial, some individuals manifested few marker lesions. In another study, IL-15-triggered NK cells were found to induce a clinical reaction in four out of six pediatric individuals with solid refractory cancers [115]. Currently, IL-15 is undergoing a trial with the infusion of NK cells for managing solid cancers as well as hematologic malignancies (NCT01385423 and NCT01875601 clinical trials). The superagonist ALT-803 (IL15N72D:IL15RαSu/IgG1 Fc complex) demonstrates a remarkable biological influence relative to that of native IL-15. This promising inducer of NK cell anti-metastatic roles is undergoing a clinical trial (NCT02099539). Moreover, genetic engineering of the ectopic expression of IL-15 is another potential approach for promoting the role of NK cells. Type I IFN is a proinflammatory cytokine that potentially preactivates NK cells, preparing them for activation by activating receptors [110]. To this end, regulatory cytokines play indispensable roles in the stimulation of NK cells for killing tumors. Numerous cytokines that activate NK cells are presently undergoing clinical or preclinical development.

## NK cell-mediated anti-tumor mechanisms

### Direct cancer killing

**Fig. 3:**
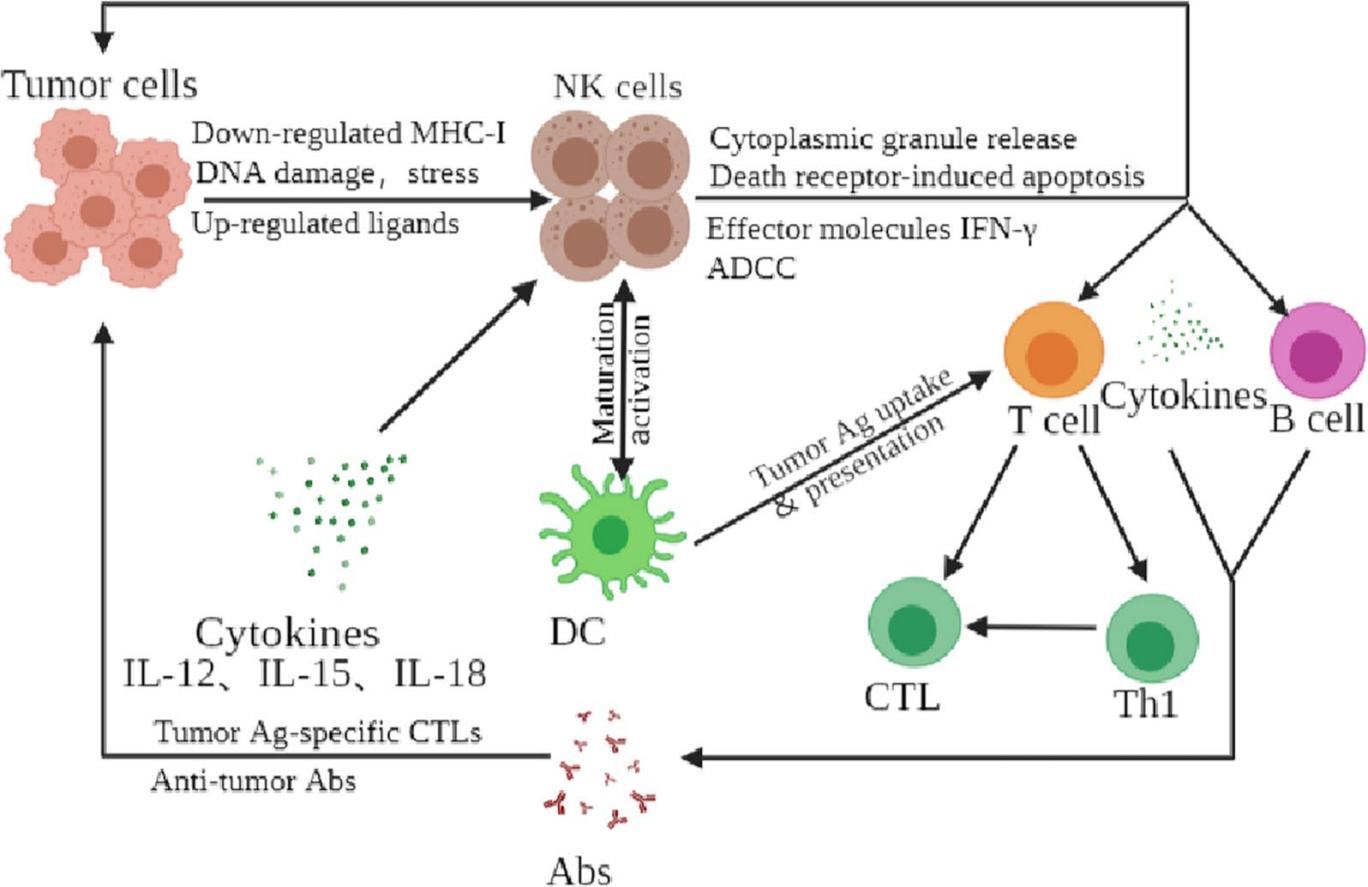
NK cells in tumor immunosurveillance. This figure shows the potential role of NK cells in tumor immunosurveillance. NK cells initially recognize tumor cells through stress or danger signals. Activated NK cells directly kill target tumor cells through at least four mechanisms: cytoplasmic granule release, death receptor-induced apoptosis, effector molecule production, or ADCC. In addition, NK cells interact as regulatory cells with dendritic cells to improve their antigen uptake and presentation and promote the generation of antigen-specific CTL responses. Also, activated NK cells induce CD8^+^ T cells to become CTLs by producing cytokines such as IFN-γ. Activated NK cells also promote CD4^+^ T cells to differentiate toward Th1 responses and promote CTL differentiation. Cytokines produced by NK cells may also regulate the production of anti-tumor antibodies by B cells. Abs, antibodies; ADCC, antibody-dependent cellular cytotoxicity; CTL, cytotoxic T lymphocyte; DC, dendritic cell; IFN, interferon; NK, natural killer

According to previous studies, NK cells have evolved multiple mechanisms for identifying healthy cells from tumor cells. Notably, NK cell killing of tumor cells is non-MHC-I- and non-antibody-dependent [13]. To escape recognition by tumor-invading cytotoxic T cells, the expression of MHC-I on the surface of tumor cells is frequently diminished or lost [13]. However, tumors that have relinquished the self-expression of MHC-I or that harbor “altered-self” stress-inducible proteins cause unregulated ligand expression on cancer cells for NK cell-stimulating receptors [13]. Therefore, because NK cells are stimulated through the initial recognition of certain “stress” or “danger” signals, there is no doubt that tumor cells are ideal NK cell targets [13]. The “self-deletion” model of NK cell recognition of tumor cells was first demonstrated through an analysis of the selective rejection of MHC-I-deficient homologous cancer cells using NK cells [116]. Additionally, the NK cell inhibitory receptors could detect this deficiency in MHC-I expression. Through their activation receptors, NK cells can kill specific MHC-I adequate cancer cells by detecting stress-triggered self-ligands [90]. In a nutshell, direct cytotoxicity mediated by NK cells exerts significant anticancer effects. NK cells kill cancer cells directly through various mechanisms, which are described as follows: (1) NK cells release the killing mediators perforin and granzyme to cause apoptosis of malignant cells, a process that requires direct contact of the NK cell recognition receptor with tumor cells; the CD56 ^low/dim^ NK cell subpopulation mainly kills target cells through this mechanism [117]. (2) NK cells trigger apoptosis through the binding of membrane TNF family molecules (FasL, TRIAL, and mTNF) to tumor cell membrane ligands. The process does not require direct contact of the NK cell recognition receptor with the tumor cell, and the CD56 ^bright^ NK cell subpopulation kills the malignant cells [57]. (3) NK cells act as a bridge between the anti-tumor antibodies IgG1 and IgG3, whereby Fab specifically recognizes the tumor while the Fc segment binds to the NK cell FcRγ IIIa to trigger antibody-dependent cell-mediated cytotoxicity (ADCC) [118]. (4) NK cells generate numerous cytokines constituting IFN-γ that exert anti-tumor effects through different mechanisms, such as the inhibition of tumor angiogenesis and activation of adaptive immune responses [119] (Fig. 3).

### Indirect cancer killing

NK cells have immunomodulatory effects, as demonstrated by their prospective effect on the functions of numerous immune cells, consisting of DCs, macrophages, T cells, and B cells [107, 120]. Furthermore, various cytokines, growth factors, and chemokines are produced through the cross-talk of these immune cells [107, 120]. The secretion of IFN-γ by stimulated NK cells triggers the transformation of CD8^+^ T cells into cytotoxic T lymphocytes (CTLs) and the specialization of CD4^+^ T cells into Th1 cells. This consequently promotes CTL differentiation [121]. NK cell-originated cytokines may also modulate anti-tumor antibody produced by B cells [122]. Additionally, cancer cells killed by NK cells can deliver cancer antigens to DCs, triggering them to maturation and presentation of antigens [123]. Activated NK cells can offer more antigenic cellular debris to other DCs through the lysis of peripheral DCs that have phagocytosed and then process exogenous antigens [123]. Thus, to promote anti-tumor immunity, activated NK cells regulate DC stimulation and maturation. These DCs potentially promote the production of antigen-distinct CTL responses as they can cross-present cancer-distinct antigens (which originated from NK cell-triggered tumor lysis) and CD8 T cells [124].

## NK cell-based therapeutic strategies

**Fig. 4:**
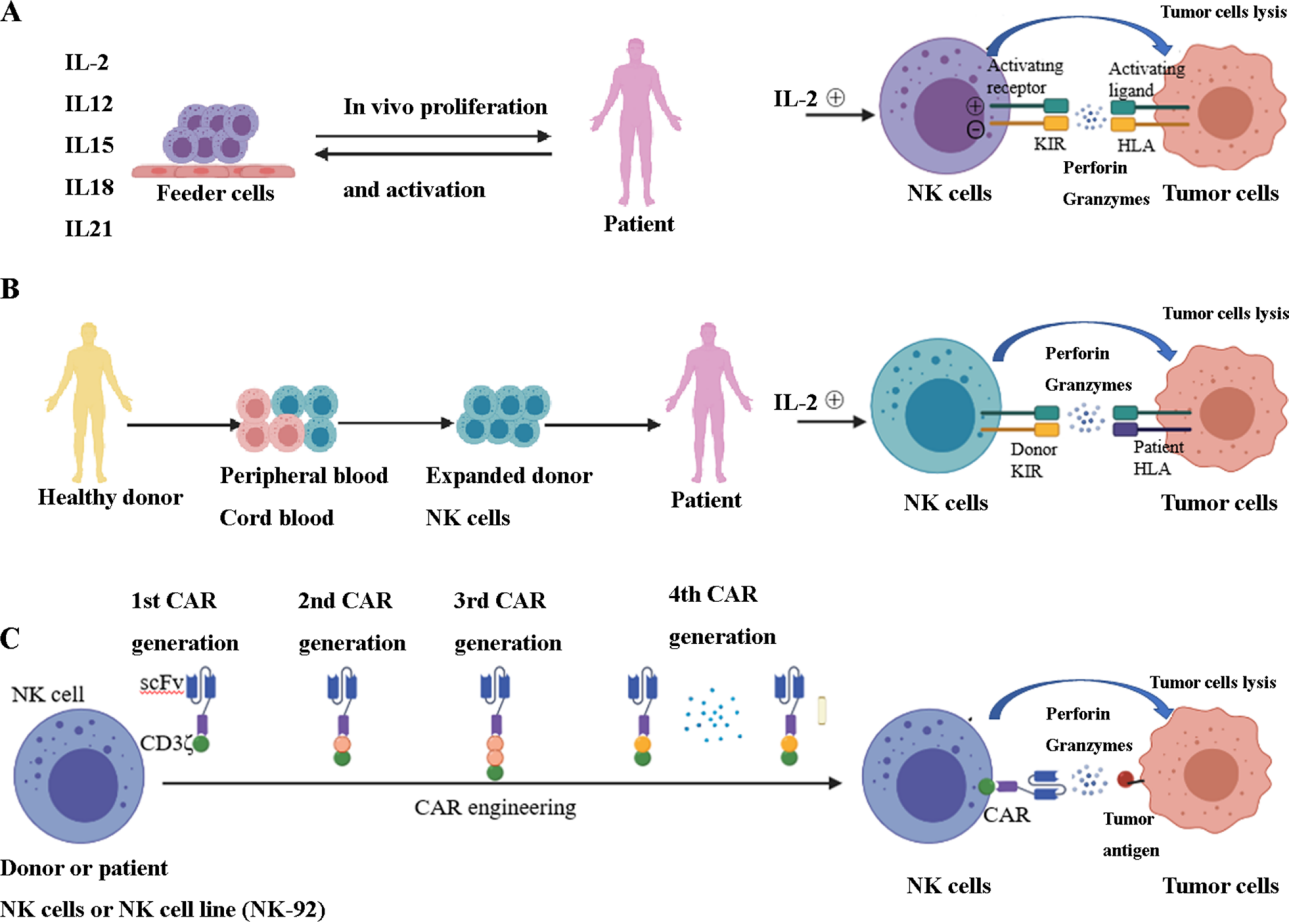
NK cell-based therapeutic strategies. **A** Autologous NK cell transfer:Cytokines IL-2, IL-12,IL-15, IL-18, as well as IL-21 in vitro can stimulate NK cells from patients’ blood and promote in vivo NK-cells proliferation and activation after NK cells are infused into cancer patients. NK cells release perforin, as well as granzyme, to cause apoptosis of tumor cells after they contact and recognize receptor on the tumor cells. **B** Allogeneic NK cell transfer: NK cells from healthy donors’ peripheral cord blood expand in vitro and are infused into cancer patients. NK cells with donor KIR release perforin, as well as granzyme, to cause apoptosis of tumor cells after they contact and recognize HLA receptor on the tumor cells. If KIR-ligand mismatch, no negative signal exists. **C** CAR-engineered NK cells: There are four different generations of CARs and they deliver stimulation signals to NK cells. Through genetic engineering modification, CAR can bind to tumor specific antigens are expressed on the surface of NK cells. After transfusion, tumor cells with specific antigens can be specifically recognized and immune responses can be triggered to achieve the purpose of tumor cell clearance

Many clinical approaches have been applied to kill cancer cells through NK cell stimulation. Cytokines, autologous as well as allogeneic NK cells, and gene-edited CAR-NK cell immune therapy are presently being pioneered in the field of NK cell treatment (Fig. 4).

### Cytokines

The cytokines IL-18, IL-2, IL-15, IL-21, and IL-12 can attenuate the immunosuppressive microenvironment in tumors by stimulating NK cell. The conflicting character of cytokines either inhibiting or activating NK cell activity disrupts this phenomenon [125]. For instance, the repressive influences of NK cells against malignant cells increase with the injection of IL‐2. However, the use of IL‐2 is minimal because of its adverse toxicity caused by the expansion of modulatory T cell populations [126]. A recent report documented that IL‐2 diphtheria toxic fusion promotes in vivo NK cell growth. Simultaneously, it cleared Treg cells. Moreover, TIM-3, a repressive checkpoint receptor, was elevated in human NK cells using IL-2, IL-21, and IL-15 alone or in combination in vitro. A recombinant fusion protein that comprised diphtheria toxin along with truncated IL-2 (IL-2DT) was designed to deplete Tregs [127]. The results demonstrated enhanced in vivo proliferation of haploid NK cells, leading to acute myeloid leukemia (AML) remission [127].

IL-15 has attracted much attention in NK cell treatment because of its beneficial properties, where regulatory T cells do not amplify but remarkably activate NK cells [128]. The cross-talk of IL-15 and its receptor generates a distinct complex that reduces the affinity of IL-2Rβ to NK cells and inhibits the activation influence of IL-2 on Treg cell expansion [129]. An IL-15 superagonist complex, namely ALT-803, was recently revealed to potentially elevate NK cells and enhance their cytotoxicity. However, in vivo as well as in vitro experiments have documented that constant inoculation of NK cells with IL-15 depletes more NK cells compared with intermittent exposure. However, ALT-803 has still yielded clinical responses in both solid tumors and hematological malignancies [130, 131]. An ALT-803 regimen given to patients in combination with PD-1 mAb reverted, with the refractory disease presenting support for the anti-tumor influence for a novel group of agents in NSCLC [130]. In a phase I first-in-human multi-center trial of ALT-803, it was found to be a safe and well-tolerated agent in study subjects who experienced relapse > 60 days post allo-HCT. It also remarkably elevated NK as well as CD8^+^ T cells along with function. This immune-activation IL-15 super-agonist should be further studied to promote anti-cancer immunity alone as well as in combination with other immunotherapies [131].

Furthermore, IL-12 can coordinate with IL-15, IL-2, and/or IL-18 to activate NK cells [132]. Prestimulating NK cells with IL-12 and activating them with IL-15 and IL-18 results in memory-like NK cells with extended survival and enhanced function. IL-12 activation requires the stimulation of STAT4, whereas the synergistic effects of IL-15, IL-18, and IL-12 do not [15]. Diminished cytotoxicity of NK cells has been documented in *IL-18*-deficient mice; however, IL-18 alone cannot sufficiently induce IFN-γ production. IL-12 alone or combined with IL-15 can be used to distinguish CD34^+^ HSC from NK cells in vitro for treatment purposes [15].

IL-21 and IL-2 synergize, causing the elevation of NKG2A, perforin, CD25, granzyme B, CD69, and CD86, and it is related to the high cytotoxicity of human NK cells [85]. IL-21 also reverses NK depletion to promote cancer regression in mice [133]. IL-21-activated NK cells modulate the amplification of intracellular pathogens such as hepatitis C virus along with *Mycobacterium tuberculosis* [15]. In a recent study, human NK cells were elevated through IL-21 along with autologous feeder cells to generate CAR-NK cells [134]. Even though research utilizing NK-92 cell lines has documented potent results, optimizing the in vitro elevation of functional NK cells in the donor or patient is essential for the generation of safe and effective CAR-NK cells.

Despite numerous clinical and translational experiments conducted to uncover more potent cytokines to trigger NK cells, research related to NK cells along with cytokines remains immature. The fundamental necessity of understanding the impact of various chemokines on the design process of NK cells warrants further exploration.

### Allogeneic and autologous NK cell treatment

Allogeneic or autologous NK cells originate from the peripheral blood [57]. They can also originate from umbilical cord blood or bone marrow as well as stimulated pluripotent or human embryonic stem cells, which are currently being explored as potential origins of NK cells with clinical significance [57]. NK cell progenitors or mature NK cells can be infused with other cells as part of the HSCT or alone following the pre-enrichment process [57].

Inhibitory receptors on donor allogeneic NK cells (e.g., KIR) do not recognize human leukocyte antigen (HLA) class I on recipient cells in case of a class mismatch. Therefore, the donor NK cells are relieved of their repressive receptor-triggered inhibition. In this case, cancer cells lack the suitable class I MHC ligands to engage the repressive KIR, and thus, they are removed by allo-reactive NK cells [13]. Numerous reports have revealed that allogeneic NK cells potentially trigger remission or suppress relapse in individuals with hematological malignancies, including AML and multiple myeloma (MM) [57, 125]. This is due to the in vitro expansion and activation of HSCT or peripatetic NK cell treatment [57, 125]. In a clinical trial of haploidentical NK cells for AML, the authors reported the induction of complete remission in dismal prognosis or elderly individuals and a 100% event-free survival rate at 18 months in a pediatric cohort [135]. Allo-reactive NK cells have the capacity to avert graft-versus-host disease (GVHD) through the elimination of host antigen-presenting cells [136]. Nevertheless, this protective influence has been challenged by a study in which allogeneic HSCT followed by the infusion of donor NK cells stimulated by IL-15 along with CD137L (ligand for co-stimulatory receptor CD137) exacerbated acute GVHD by promoting the underlying T cell allogeneic response [137]. Differences in the origin as well as development of the infused NK cells may explain these contradictory findings. Indeed, inadequate T cell depletion in KIR-mismatched grafts may cause severe GVHD and offset the clinical benefit of allogeneic NK cells [138]. Collectively, further studies are warranted to explore the discrete conditions and types of cancers that would benefit from allogeneic NK cell infusion.

In individuals with hematological cancer undergoing autologous HSCT, the number of blood NK cells recovers early after transplantation. Several NK cells were linked to positive results in these patients, which illustrated the anti-cancer ability of NK cells [139, 140]. Other reports have indicated that autologous NK cell expansion and infusion in individuals with metastatic melanoma, advanced gastrointestinal cancer, or renal cell carcinoma do not translate into a clinical response [141, 142]. Notably, NK cells that persist in the circulation from a secondary infusion cannot kill tumor cells unless they are restimulated in vitro. This finding highlights the need for combinatorial approaches to fully exploit the ability of autologous NK cells [108].

In conclusion, both allogeneic and autologous NK cell therapy have demonstrated clinical efficacy either alone or in combination with conventional therapies. Table 3 summarizes the findings of clinical trials where NK cells have been infused into cancer patients. There is ongoing research on autologous NK cells and some 50 clinical trials are presently underway. Furthermore, studies on the efficacy of allogeneic NK cell transfer are presently underway and exhibiting promising clinical potential (e.g., NCT00720785, NCT03068819, NCT02782546, NCT01898793, NCT03081780, NCT03319459, NCT03213964, NCT03019640, NCT01729091, NCT01787474, NCT02809092, NCT02271711, and NCT03579927). Adoptive cell treatment with allogeneic NK cells has similar disadvantages to autologous NK cells, such as timely ex vivo expansion as well as the activation of clinical-grade NK cells and in vivo persistence after infusion. In the future, scientists should invest more effort in this area to refine NK cell treatments.

**Table 3:** Clinical trials of NK cells in cancer

Cancer type	Source	Enrichment of NK cells	Lymphodepletion	Patient (N)	Clinical response
AML, CML, MDS	Haploidentical (HSTC donor)	CD3^−^CD56^+^ selection	None	5	CR in 4 [141] patients
AML	Haploidentical	CD3 depleted PBMCs, IL-2 stimulation	Flu/Cy	19	CR in 5 patients [142]
AML	Haploidentical	CD3 depleted PBMCs, IL-2 stimulation (n = 32) CD3 depleted PBMCs, CD56 selection, IL-2 stimulation (n = 10)	Flu/Cy	42	CR in 9 patients [125]
AML	Haploidentical	CD3 depleted PBMCs (with or without CD56 selection), or CD3 and CD19 depleted PBMCs, IL-2 stimulation		15	CR in 8 patients [125]
AML	Haploidentical	CD3 and CD19 depleted PBMCs, IL-15 stimulation	Flu/Cy	40	CR in 7 patients [143]
AML	Umbilical cord blood	Differentiation and expansion from CD34^+^ cells	Flu/Cy	10	CR in 10 patients[144]
AML, CML	HSCT donor	CD3 depletion, co-culture with K562-mbIL-21	HSCT conditioning	13	CR in 7 of 8patients with AML and in all 5patients with CML [145]
MM	Autologous or haploidentical	Co-culture with K562-mbIL-15-4-1BBL, CD3 depletion	Bortezomib alone or with Flu/Cy and dexamethasone	7	Two patients were treatment-free for 6 months [146]
B-NHL	Haploidentical	CD3and CD19depleted PBMCs, IL-2 stimulation, pretreatment with rituximab	Flu/Cy, methylprednisolone	14	CR in 2 patients; PR in 2 patients [147]
Neuroblastoma	Haploidentical	CD3^−^CD56^+^selection, IL-2 stimulation, anti-GD2 after NK cell infusion	Cy, vincristine, and topotecan	35	CR in 5patients; PR in 5patients [148]
RCC	Haploidentical	CD3depleted PBMCs, IL-2 stimulation	Flu	7	No[142]
Melanoma, RCC	Haploidentical	CD3-depleted PBMCs, IL-2 stimulation	Cy and methylprednisolone	16	SD in 6patients[142]
Ovarian cancer, breast cancer	Haploidentical	CD3-depleted PBMCs	Flu/Cy, TBI (2 Gy)	20	PR in 4patients; SD in 12patients [149]

### CAR-NK cell therapy

#### Background

The recent approval by the U.S. Food and Drug Administration (FDA) of CAR-T cell therapy targeting CD19 is a critical breakthrough in the design of genetically modified cell treatments for cancer. This has also stimulated much attention in the preparation of CAR-NK cells for tumor immune therapy [143]. In clinical research, the transfusion of unmodified allogeneic NK cells has demonstrated slight efficacy, specifically in AML. However, their short lifespan (2–3 weeks after transfusion) has somewhat limited their success. Allogeneic NK cells, despite their ability to exert allo-reactivity, do not cause acute GVHD. Hence, relative to CAR-T cells, allogeneic NK cells are potential therapeutic agents that require no other genetic modifications. Thus, the novel biological properties of NK cells render them a more attractive origin of genetically modified immune cell-based immune therapy.

#### Study overview

Progress in research has contributed to a gradual improvement in the design of CARs. Several generations of CARs now exist: 1st-generation CARs comprise a basic structure with one signaling region [144]; 2nd-generation CARs harbor an extra co-stimulatory domain, such as CD28 or 4-1BB [145, 146]; 3rd-generation CARs have multiple co-stimulatory domains [147, 148]; and 4th-generation CARs have multiple co-stimulatory domains and cytokines signals (Fig. 4C). Many co-stimulatory domains have been explored, consisting of immune globulin superfamily members (CD28 and ICOS), TNF receptor superfamily members (4-1BB, CD27, OX40, and CD40), and others (e.g., CD40L and TLR) [149]. At the very beginning, the CAR constructs used for CAR-NK cells were optimized for T-cell signaling and function. Although certain signaling/co-stimulatory domains used in CAR design (e.g., CD3ζ and 4-1BB) are shared between T and NK cells, the role of other co-stimulatory molecules (e.g., CD28) in NK cells is elusive [150]. To date, numerous reports have been published on the greater specificity for NK-cell signaling, DAP10, DAP12, and 2B4 [151, 152]. DAP10 and DAP12 are activation motifs harboring immunoreceptor tyrosine-based activation and deliver stimulation signals to NK cells. DAP10 signals the activating receptor NKG2D, whereas DAP12 mediates signaling via NKG2C, NKp44, and the activating killer immunoglobulin receptor (KIR). Another activating receptor is 2B4, which belongs of signaling lymphocyte activating molecule (SLAM) family; when bound to its natural ligand CD48, 2B4 recruits articulator molecules such as SLAM-associated protein (SAP) (ITSM) to mediate signal transduction via its immunoreceptor tyrosine-based switch motif [153]. More recently, 4^th^-generation CARs were investigated [154]. These vectors incorporate transgenic “payloads” designed to promote the growth, persistence, and anti-tumor activity of CAR-engineered NK cells. To date, results have been published that justify the superior activity of these latest CAR design revisions, and the expansion the function of CAR-modified NK cells within the existing approach to cancer immunotherapy is promising [155, 156].

#### NK cell sources

In addition, scientists have explored different cell origins for the production of CAR-expressing NK cells. These cell sources include peripheral blood [157–159], umbilical cord blood [134, 156, 160–163], stem cells (including hematopoietic and stimulated pluripotent stem cells) [66, 164, 165], and NK cell lines [162, 166, 167]. Notably, each source has its unique advantages and disadvantages (Table 4).

**Table 4:** Sources of NK cells and their unique advantages and disadvantages

Source	Advantages	disadvantages
PB	Mature phenotype Highly functional and cytotoxic	Only 5%–10% of PB lymphocytes are NK cells Heterogenous product Not readily available, need donors
CB	Readily available from global CB banks.15%–30% of CB lymphocytes are NK cells. Transcriptomic profile supports high proliferative potential	Numerically few and therefore requires ex vivo expansion Heterogeneous product
iPSC	High proliferative capacity Homogeneous product	Immature phenotype Low ADCC due to low CD16 expression Long culture condition
NK-92 cell line	High proliferative capacity Easy to manipulate and engineer Homogeneous product Reduced sensitivity to freeze/thaw cycles	Derived from a patient with NK lymphoma Need for irradiation Limited in vivo persistence following irradiation Low ADCC due to low or absent CD16 expression

#### Clinical efficacy of CAR-NK in cancers

Currently, CAR-NK cell therapy has yielded preclinical anti-malignant activity both in hematological cancers and solid tumors, including leukemia, lymphoma, myeloma, ovarian cancer, and glioblastoma [168]. In some clinical trials, this therapy has exhibited efficacy.

##### AML

In 2018, *Jianhua Yu’s* group reported a phase I first-in-human clinical trial involving CD33-CAR NK cells for individuals with relapsed as well as refractory AML [169]. In their study of CD33-CAR-NK cells, they enrolled three patients and tested their safety, but no adverse events were reported. Notably, the author revealed that CAR NK-92 cells could be generated at a much lower cost than CAR-T cells, and believed that even after optimization they would be broadly accessible for cancer management [169].

##### Lymphoma

In 2020, Rezvani et al. demonstrated that allogeneic CAR-NK cells originated from cord blood were beneficial for high-risk B cell lymphoma and CD19^+^ CLL [156]. The use of CAR-NK cells exhibited no association with the onset of cytokine release syndrome, neurotoxicity, or GVHD. Furthermore, the quantities of inflammatory cytokines, comprising IL-6, did not exceed baseline levels and the maximum tolerated dose was not attained. Eight (73%) of the 11 patients treated responded, of which seven patients (four with lymphoma as well as three with CLL) were in complete remission, whereas one individual had Richter transformation in partial remission but persistent CLL. Their reactions were prompt and were reported for 30 days post-infusion for all dose levels. Infused CAR-NK cells were expanded and then persisted at low quantities for at least one year [156]. The company Fate Therapeutics recently announced clinical data on FT596, an allogeneic iPSC-derived CAR-NK cell. FT596 monotherapy demonstrated durable tumor clearance and extended in vivo survival, demonstrating the promise of iPSC-derived NK cell therapy as a novel cancer immunotherapy for development [170]. In addition, FT596 exhibited enhanced killing of CD20^+^ lymphoma cells in vivo when combined with rituximab compared with rituximab alone. This is a preliminary indication that the combination of antibody drugs and NK cell therapy can have a synergistic effect and is another new direction for CAR-NK cell development [170].

##### MM

MM is a malignancy caused by genetic mutations that occur during the differentiation of B lymphocytes into plasma cells. Common tumor antigens in MM cell lines include cell surface glycoprotein CD2 subset 1 (CS1) [171], CD138 [172], and BCMA [173]. Relatively little research has been conducted on CAR-NK cell products for the treatment of MM. Most of the CAR-NK cell products currently used to treat MM use the NK92 cell line, and the tumor antigens used are mostly CS1 [174] and CD138 [175]. CS1 is a highly expressed protein on the surface of MM cells and is mostly involved in MM cell adhesion and growth [174]. CD138 is involved in the adhesion, growth, and maturation of MM cells and is a major diagnostic marker for MM. In vitro studies have demonstrated that CS1-CAR-NK92 cells and CD138-IFNα-CAR-NK92 cells, designed using CS1 and CD138 as targets, respectively, successfully inhibited MM cell growth and prolonged the survival of myeloma mice [174].

##### Solid tumors

CAR-T cells are subject to PD-1/PD-L1-mediated immunosuppression in the fight against solid tumors. NK cells with very low surface PD-1 expression and relatively little immunosuppression by the tumor microenvironment may be good candidates for fighting solid tumors. In solid tumor research, CAR-NK cells are mostly targeted at metastatic solid malignancies expressing tumor-associated antigens such as HER2, PSMA, mesothelin, ROBO1, or MUC1, including prostate cancer, ovarian cancer, breast cancer, pancreatic cancer and non-small-cell lung cancer. In addition, PD-L1 is upregulated in the TME and immunosuppressive cells in several cancer types. A new NK-92 cell line was designed to target PD-L1, ER-retained IL-2, and a high-affinity CD16 CAR called PD-L1-targeted haNK (t-haNK). Exciting preclinical data suggest that these cells have specific anti-tumor effects against 15 tumor cell lines in vitro and strong anti-tumor effects against triple negative breast, bladder, and lung cancers in vivo. The QUITL3.064 phase I clinical trial (NCT04050709) is currently underway with PD-L1 t-haNK in combination with other agents for assessing safety and efficacy in patients with locally advanced or metastatic pancreatic cancer (NCT0439099).

Given the safety and efficacy of CAR-NK cell therapy, numerous clinical trials on hematologic cancers and solid tumors are currently underway. Table 5 presents CAR-NK cell-based therapy clinical trials for hematologic malignancies and solid tumors.

**Table 5:** The clinical trials of CAR-NK cell-based therapy for hematological malignancies and solid tumors

Cancer type	ClinicalTrials.gov Identifier	Initial time	Phase	N	Primary study endpoint
R/R Non-Hodgkin Lymphoma	NCT04639739 CD19	December 17, 2020	Phase I	9	Incidence of dose limiting toxicity Incidence and severity of AEs and SAEs
Relapsed and Refractory B Cell Lymphoma	NCT03692767 CD22	March 2019	Phase I	9	Occurrence of treatment related adverse events as assessed by CTCAE v4.0
Relapsed and Refractory B Cell Lymphoma	NCT03690310 CD19	March 2019	Phase I	9	Occurrence of treatment related adverse events as assessed by CTCAE v4.0
Epithelial Ovarian Cancer	NCT03692637 Mesothelin	March 2019	Phase I	30	Occurrence of treatment related adverse events as assessed by CTCAE v4.0
metastatic Solid Tumours	NCT03415100 NKG2D-ligand	January 2, 2018	Phase I	30	Number of Adverse Events
Castration-Resistant Prostate Cancer	NCT03692663 PSMA	December 2018	Phase I	9	Occurrence of treatment related adverse events as assessed by CTCAE v4.0
Solid Tumors	NCT03940820 ROBO1	May 2019	Phase I/II	20	Occurrence of treatment related adverse events as assessed by CTCAE v4.03
Relapse/Refractory MM	NCT03940833 BCMA	May 2019	Phase I/II	20	Occurrence of treatment related adverse events as assessed by CTCAE v4.03
Recurrent/Metastatic Gastric or Head and Neck Cancer	NCT04847466 Irradiated PD-L1	April 22, 2021	Phase II	55	ORR
Relapsed and Refractory B Cell Lymphoma	NCT03824964 CD19/CD22	February 1, 2019	Phase I	10	Occurrence of treatment related adverse events as assessed by CTCAE v4.0
B Lymphoid Malignancies	NCT04796675 CD19	April 10, 2021	Phase I	27	Incidence of Treatment-related Adverse Events
Relapsed/Refractory CD33 + AML	NCT02944162 CD33	October 2016	Phase I/II	10	Adverse events attributed to the administration of the anti-CD33 CAR-NK cells
CD19 Positive Leukemia and Lymphoma	NCT02892695 CD19	September 2016	Phase I	10	Adverse events attributed to the administration of the anti-CD19 CAR-NK cells
Pancreatic Cancer	NCT03941457 ROBO1	May 2019	Phase I	9	Occurrence of treatment related adverse events as assessed by CTCAE v4.03
CD19 + Relapsed/Refractory Hematological Malignancies	NCT04796688 CD19	March 10, 2021	Phase I	27	Incidence of Treatment-related Adverse Events
Relapsed/Refractory B-Lymphoid Malignancies	NCT03056339 CD19	June 21, 2017	Phase I/II	36	Toxicity and efficacy

##### Advantages and challenges of CAR-NK cells

NK cells have some powerful therapeutic advantages. (1) They are more widely available, can be derived from allogeneic cells, and do not need to rely on the patient’s own specific immune cells. (2) NK cells do not require MHC molecules for antigen presentation or antigen activation and can target a wide range of pathogenic antigens with greater cytotoxicity. (3) NK cells do not secrete the major cytokines that trigger CRS and can greatly mitigate the risk of adverse effects. (4) Allogeneic NK cells also do not cause graft-versus-host reactions. However, CAR-NK cell therapy still entails some difficulties and dilemmas. (1) In vitro expansion of NK cells is the first hurdle for CAR-NK cell immunotherapy. The number of NK cells from a single donor is not sufficient for therapy, which makes the expansion and activation of NK cells critical. This production process usually takes two to three weeks to culture NK cells. Therefore, obtaining enough NK cells remains a challenge. (2) Selecting the appropriate method for transducing CARs into NK cells is the key to CAR-NK cell immunotherapy [57]. Thus far, both viral and nonviral vectors have been used to transform CARs. Although retroviral vectors have high transfection efficiency, they may cause insertional mutations, carcinogenesis, and other adverse effects. By contrast, lentiviral vectors, despite exhibiting a low incidence of insertional mutations, have transfection efficiencies as low as 20% for peripheral blood NK cells [57]. The transfection of CAR-NK cells with mRNA is also considered a safe and practical transfection method. A study demonstrated that in xenograft tumor models, mRNA-transfected NK cells exhibit significant cytotoxicity after 24 h of electroporation, with receptor expression levels exceeding 80% [57]. Furthermore, it has recently been demonstrated that the transfection of mRNA can effectively avoid “targeted non-tumor” toxicity, which is a critical factor limiting the clinical application of CAR-modified immunotherapy. However, the anti-tumor effect of CAR-NK cells transfected with mRNA through electroporation is transient as the expression level of CARs does not exceed 3 days. (3) Another challenge for CAR-NK therapy is the impact of the TME. (4) The primary barrier to reliable preclinical evaluation of solid cancer CAR-NK therapy is the lack of clinically relevant models of animals that encapsulate the complexity of interactions in the TME [168]. Most studies have relied on human tumor cell lines derived from immunocompromised NOD scid γ null (NSG) mice, which lack an effective immune system [168]. While existing NSG models can be applied to rapidly assess the function and persistence of CAR effector, they cannot establish a clinically relevant TME or accurately estimate CAR-NK cell function and persistence. Furthermore, such models can explore the cross-talk between different immune cells and tumors.

### NK cell-based immune checkpoint

#### KIRs

The KIR family (also known as CD158) is a diverse and polymorphic group of NK cell receptor subtypes containing both inhibitory and activating KIRs, each of which recognizes a specific HLA class I congener (HLA-A, -B, or -C) as a ligand [176]. IPH2101 and lirilumab (IPH2102/BMS-986015) are IgG4 mAbs against the KIR2DL1/2/3 NK cell inhibitory receptor, while IPH4102 is a humanized anti-KIR3DL2 IgG1 mAb. The IPH2101 blockade of KIR improves survival in vivo, and preclinical evidence also suggests its effectiveness in AML cells [177]. A phase I study of IPH2101 in elderly patients with AML in first complete remission demonstrated that the overall and relapse-free survival compared favorably with reports in comparable patient populations [177]. Phase II trial results of lirilumab indicated poor efficacy of monotherapy for MM [178]. However, lirilumab in combination with full-dose azacitidine was well-tolerated in patients with heavily pretreated/relapsed AML [179]. A recent study reported the efficacy and tolerability of lirilumab as a single agent or in combination with azacitidine in patients with myelodysplastic syndrome (MDS) [180]. IPH4102, also known as lacutamab, was well-tolerated in a phase I clinical evaluation in relapsed/refractory cutaneous T-cell lymphoma, with the most common adverse effects including edema, fatigue, and lymphopenia [181]. The clinical activity was also encouraging, with ORR achieved in 16 of 44 patients (36%) [181]. Patients with relapsed/refractory cutaneous T-cell lymphoma with Sézary syndrome exhibited a better clinical response (43%) [181].

Several clinical trials are still ongoing in cisplatin-ineligible muscle-invasive bladder cancer (NCT03532451), relapsed or refractory tumors (NCT02813135), locoregionally recurrent squamous cell carcinoma of the head and neck (NCT03341936), and advanced T cell lymphoma (NCT03902184).

#### NGK2A and CD94

NKG2A (also known as CD159) and CD94 are heterodimeric inhibitory receptors of the C-type lectin family, which recognize the nonclassical MHC-I molecule HLA-E as a ligand [182]. The results of in vitro and in vivo studies have suggested that humanized anti-NKG2A/CD94 (IPH2201, monalizumab) antibodies are safe and effective for use in hematological malignancies and solid tumors [183]. In in-vitro trials, monalizumab improved NK cell dysfunction in chronic lymphocytic leukemia [184]. Monalizumab was well-tolerated as a single agent for the treatment of gynecologic malignancies (up to 10 mg/kg administered intravenously or by SC) [185]. A preliminary evaluation of the safety and efficacy of monalizumab in combination with cetuximab in previously treated, recurrent, and/or metastatic squamous cell carcinoma (SCC) of the head and neck revealed an ORR of 27.5%, a median PFS of 5 months, and a median overall survival (OS) of 10 months with the combination [186]. This is an encouraging result when compared with the historical record of cetuximab efficacy alone in previous studies (ORR 12.6%, PFS 2.3 m, OS 5.6 m). The combination therapy had similar adverse effects to cetuximab alone. Overall, blocking NKG2A/CD94 represents an exciting therapeutic approach; in particular, its combination with other immuno-oncology therapeutics is the way forward and warrants further exploration. In addition, clinical trials are currently evaluating the efficacy of monalizumab in combination with a variety of other targeted agents for the treatment of multiple tumors (NCT02643550, NCT02671435, NCT03822351, NCT03833440, and NCT03088059).

#### TIGIT and CD96

TIGIT is an immunosuppressive receptor expressed on NK and T cells [187]. CD96 belongs to the same immunoglobulin superfamily as TIGIT and has similar inhibitory effects, but it binds with lower affinity to CD155, its ligand. CD155 (mainly) and CD112 act as ligands for TIGIT and CD96 binding to suppress T-cell- and NK-cell-mediated immunity [188]. It is hardly expressed in normal human tissues, but many tumor cell lines and primary malignancies highly express CD155 [189–191]. Among the functions of CD155, immunomodulation through its interaction with the inhibitory receptors TIGIT and CD96 and the activating receptor CD226 is of particular interest. Various cancers exhibit upregulation of CD155 and corresponding upregulation of NK and T cell expression of TIGIT and CD96 to evade anti-tumor immunity through inducing T cell or NK cell suppression [189–191].

Animal experiments revealed that TIGIT intrinsic expression inhibits NK and CD8^+^ T cell function, thereby aiding colorectal cancer cell growth in vivo [192]. TIGIT is associated with NK cell depletion in tumor-bearing mice and patients with colon cancer, and this depletion is restored by its blockade, thereby stimulating strong anti-tumor immunity [187]. The presence of NK cells is crucial for the therapeutic efficacy of both checkpoints of TIGIT and/or PD-L1 blockade or dual blockade, as NK cell deficiency is associated with a lower frequency of IFN-γ or TNF-secreting TIL (CD8^+^) and a higher frequency of PD-1-expressing TIL (CD8^+^) [187]. NK cells account for 25–50% of hepatic lymphocytes, which indicates their importance for liver immunity. Furthermore, the survival and prognosis of patients with hepatocellular carcinoma (HCC) are positively correlated with the number of NK cells in blood and tumor tissue [193, 194]. Cancer progression in HCC patients is associated with dysfunctional NK cell infiltration, mainly in the CD11b^−^CD27^−^ subpopulation [193, 194]. Sun et al. identified depleted tumor-infiltrating CD96^+^ NK cells and found that their expression was correlated with poor clinical outcomes in HCC patients [195]. NK cell depletion was reversed when CD96–CD155 interactions or TGF-β1 were blocked [195].

In recent years, the combination of checkpoint inhibitors has received increasing attention for achieving synergistic effects. Enhancing CD8^+^ T-cell activation has been reported to improve the survival rate of hormonal mice with dual targeting of PD-1 and TIGIT. Dixon et al. reported that dual blockade of TIGIT and PD-1 produced a synergistic anti-tumor effect leading to complete tumor regression in the MC38 colon cancer model [196]. In melanoma patients, dual blockade of TIGIT and PD-1 synergistically increased tumor infiltration and tumor antigen-specific CD8^+^ T cell proliferation, degranulation, and cytokine secretion, indicating the potential for dual blockade [197]. Hong et al*.* suggested that PD-1 and TIGIT could also be potential targets for the treatment of RCC [198]. In patients with GBM, this dual blocker also improved anti-tumor immunity and survival [199]. While these studies reflect the efficacy of double checkpoint blockade in various cancers by exploring the role of T cells, some studies have also suggested that the efficacy of the double checkpoint is also dependent on NK cells. Anti-TIGIT plus anti-PD-L1 blocker prevented NK cell depletion in hormonal mice and colon cancer patients [187]. On the other hand, anti-CD96 combined with adriamycin chemotherapy, anti-CTLA-4, or anti-PD-1 exhibited more effective inhibition of tumor metastasis in three different tumor models [200]. Bladder cancer patients with failing NK cells exhibited upregulation of TIM-3 and TIGIT both in the periphery and in the tumor [201]. Indeed, the roles of TIGIT and CD96 in NK cell depletion in various cancers are still under investigation, and further revelations are required to determine their potential as monotherapy or in combination with other checkpoints. Clinical trials are ongoing regarding TIGIT blockade as a monotherapy or in combination with other therapies for treating various hematological malignancies and solid tumors (e.g., NCT04818619, NCT04150965, NCT04656535, NCT04500678, NCT04732494, NCT0435383, NCT04693234, NCT04570839, NCT04354246, NCT04047862, NCT04952597, NCT04457778, NCT04543617, NCT03563716, and NCT04746924). Overall, TIGIT monoclonal antibody is in the early stage of clinical trials, and the results are subject to further observation.

## Future perspectives

Many strategies have been developed for exploiting the anti-tumor properties of NK cells. Researchers are testing IL-2 and IL-15, two cytokines that promote NK cell activity, but exacerbating the immune response poses a safety concern. NK cells from healthy donors are stimulated in vitro by IL-2 and IL-15 and then transfused back into the blood of cancer patients. This strategy can take advantage of the mismatch between donor KIR and patient HLA to reduce the suppression of NK cell function and promote their anti-tumor activity. Since the introduction of CAR engineering technologies, the paradigm guiding the field of cell therapy has shifted. As the first immune effector cells engineered by CAR with promising results in the clinic, CAR-T cells have set the pace for the future design of CAR-based immunotherapy. NK cells characterize a specialized population of immune effector cells with a rapid response and powerful anti-tumor capacity. Despite their success, CAR-T cells still have significant drawbacks that have fueled research into other immune effector cells as an alternate approach for CAR engineering. In the past decade, clinical research on hematological cancers has pioneered the concept of peripatetic NK cell immunotherapy. Evidence suggests that NK cells have high safety and efficacy. Some clinical efficacy has also been demonstrated for allogeneic as well as autologous NK cell therapy, either alone or in combination with conventional therapies. Crucially, tumor antigen-expressing CAR-NK cell therapy increases anti-tumor activities. Thus, NK cell transfer presents an effective method of fighting cancer. In addition, antibodies that directly target NK cell inhibitory receptors, such as those targeting KIRs, NKG2A, and TIGIT, can enhance NK cell responses and thus kill tumor cells, and some are currently being validated in clinical trials. Therefore, based on the pan-specific recognition property of NK cells, NK cell-based multiple immune combination therapy is a strategy for further improving anti-tumor efficacy and deserves further exploration.

## Data Availability

Not applicable.

## Abbreviations

NK cells: Natural killer cells
SCC: Squamous cell carcinoma
OS: Overall survival time
HCC: Hepatocellular carcinoma
MDS: Myelodysplastic syndrome
SLAM: Signaling lymphocyte activating molecule
DCs: Dendritic cells
TME: Tumor microenvironment
TLR: Toll-like receptor
mAbs: Monoclonal antibodies
CLPs: Common lymphocyte progenitors
LNs: Lymph nodes
PB: Peripheral blood
